# Patients' experiences of medication management while navigating ongoing care between outpatient services: A qualitative case study of patients on hemodialysis

**DOI:** 10.1016/j.rcsop.2024.100418

**Published:** 2024-02-08

**Authors:** Tracy Zhang, Mai Mohsen, Angelina Abbaticchio, Marisa Battistella

**Affiliations:** a Leslie Dan Faculty of Pharmacy, University of Toronto, Ontario, Canada; b Department of Nephrology, Toronto General Hospital – University Health Network, Ontario, Canada

**Keywords:** Hemodialysis, Medication management, Care navigation, Outpatient, Interprofessional collaboration, Patient-centered care

## Abstract

**Background:**

Patients on hemodialysis have complex medical diagnoses and medication regimens, requiring access to numerous health services and consultation with various healthcare providers. While interprofessional collaboration can optimize care among hemodialysis patients, these patients commonly experience medication-related problems and frequent hospitalizations resulting from miscommunications and mismanagement of medications.

**Objectives:**

This study aims to capture the lived experiences of patients on hemodialysis to reveal their medication management needs as they navigate ongoing care between various outpatient services.

**Methods:**

A qualitative methodology was used to explore the perspectives of hemodialysis patients. One-on-one, in-person, semi-structured interviews were conducted at an outpatient hemodialysis clinic located inside an urban teaching hospital. English-speaking adults 18 years and older who have been followed at the clinic for at least three months were selected through random, convenience sampling. Interviews were recorded and transcribed verbatim. Patients were recruited and data were collected iteratively and continued until data saturation was reached. Data was analyzed through the lens of the Picker Principles of Patient Centered Care using a general inductive approach.

**Results:**

A total of nine interviews were conducted. Two major themes, medication management and care navigation, were identified. Though patients had a wealth of knowledge about their medications, and they were motivated to self-manage their medications to enhance their well-being, they experienced barriers with medication management. Patients further expressed challenges with navigating care and spoke of the importance of having good rapport with healthcare providers who are attentive to their needs.

**Conclusions:**

The results revealed a need for improved support for self-care and interprofessional collaboration to possibly reduce the burden of medications and care fragmentation experienced by patients and improve continuity of care for patients.

## Introduction

The number of Canadians receiving chronic dialysis nearly doubled from 11,601 in 2000 to 23,125 in 2019.^1^ More than three-quarters of these patients receive hemodialysis in institutions, often in an ambulatory clinic where they undergo three to four-hour treatments three to four times per week. In addition to hemodialysis, patients with end stage kidney disease (ESKD) often have multiple medical comorbidities, requiring them to manage complex medication regimens with an average pill burden of 19 pills per day.^2^ Medications often come with different instructions to take before or after dialysis and may change frequently depending on routine laboratory tests. Management of such complex, frequently changing medication regimens can increase the risk of drug therapy problems, defined as any undesirable event that a patient may experience involving medications, including taking a medication that is no longer required, not taking a medication that is required, or experiencing side effects or drug-drug interactions. If left unresolved, drug therapy problems can contribute to poor health outcomes.^3^

Patients on hemodialysis consult numerous health services, such as wound care, and chiropody, and attend regular follow-up appointments with various healthcare providers (HCPs), including nephrologists, family physicians, and pharmacists, in part to ensure their medication regimes are safe and effective. For example, a patient's nephrologist may adjust their medications to ensure proper electrolyte balance, their family doctor may prescribe a new antibiotic for an infection, and they may visit their pharmacist to learn how and when to take medications. Within hemodialysis clinics and across transitions of care, medication errors, particularly medication omissions, are common.^4^^,^^5^ A 2008 study showed that when patients on hemodialysis moved from an in-patient dialysis setting to an outpatient setting, 78% had at least one unintended variance in their medications occur.^6^ Thus, as ambulatory patients on hemodialysis navigate complex, outpatient transitions of care and often receive medication recommendations from different healthcare providers in various settings, they are at risk of unintended variance, miscommunications and other medication-related errors.

While existing studies describe similar medication-related challenges for patients on hemodialysis, including taking numerous medications, navigating multiple transitions of care, and receiving care from so many different HCPs, few solutions to these challenges have been studied and implemented.^7^^,^^8^ The care that patients on hemodialysis receive is often fragmented due to there being multiple care areas, different institutions, and numerous individuals involved, making it difficult to study the effectiveness of interventions.

Given that patients on hemodialysis encounter many HCPs, one area that could be further studied to provide insight on how to reduce medication-related harm is the role of interprofessional collaboration. According to the World Health Organization, interprofessional collaboration strengthens health systems and improves health outcomes.^9^ Since the publication of their Framework for Action on Interprofessional Education and Collaborative Practice, many studies have examined the dynamics and impact of different healthcare professionals working together.^10^, ^11^, ^12^ Optimizing the care of the patient is often considered an intention of effective interprofessional collaboration. Despite calls to action to improve interprofessional collaboration in healthcare, many patients continue to experience poor outcomes. An American study showed that each patient on hemodialysis still experiences an average of 4.5 medication-related problems,^13^ and Canadian hemodialysis patients are hospitalized an average of 1.1 times per patient per year.^2^ This may be due to the lack of patient-centeredness within studies of interprofessional collaboration.

While patients are cited as important members of the interprofessional team, studies tend to focus on HCP experiences and various tools to enhance how HCPs practice collaboratively with one another and less on the patient perspective.^14^ Health Canada's Institute for Healthcare Improvement has emphasized the importance of including patient perspectives in assessments of quality of care and health outcomes to improve population health and reduce the per capita cost of healthcare.^15^ To understand the nuances of managing complex medications and navigating a fragmented healthcare system, there is a need to closely examine the experiences of patients on hemodialysis through the use of qualitative research methodology. This approach is intended to facilitate an in-depth understanding of patient experiences, and may reveal ways to enhance patient-centered care (PCC) in regard to medication management, potentially helping to reduce medication-related problems.

## Theory

PCC is a proposed way of clinical practice that places patients at the center of their own healthcare.^16^ This approach accounts for individuals' preferences, needs and values to guide clinical decisions.^17^ The Institute of Medicine states that PCC “makes the patient an integral part of the care team who collaborates with care providers in making clinical decisions”.^18^ PCC is associated with improved well-being, satisfaction with care and health outcomes among complex patients.^19^^,^^20^ Integrating principles of PCC into the management of complex medication regimens can promote meaningful collaboration between HCPs and patients.

One of the most prominent international frameworks used to describe PCC and patient experience is the Picker Principles of Patient Centered Care. Through extensive focus groups with patients, their families and various healthcare professionals, The Picker Institute, The Commonwealth Fund, and a group of researchers from Harvard Medical School identified eight dimensions of PCC.^21^^,^^22^ The eight Picker Principles of Patient Centered Care discuss the importance of respect for patients, their preferences and taking a holistic approach, appreciating patients as individuals for care to truly be compassionate. These principles also cover the need for clear information, continuity of care and the importance of effective and timely treatment by trusted healthcare professionals.^21^ Using these dimensions, the Picker Institute has created numerous large-scale and impactful surveys globally to understand patient experiences of care^11^ including the National Cancer Patient Experience Survey with NHS England in 2021 and the Lymphoma Coalition Global Patient Survey in 2020.^23^^,^^24^

## Material and methods

The research team conducted a qualitative case study using one-on-one, in-person, semi-structured interviews. Case study was selected as the methodology to examine the in-depth, lived experiences of a complex group of patients.^25^ While case studies may sometimes refer to individuals as one bounded case, this study considers the bounded case as the unique group of patients who receive outpatient hemodialysis at a clinic located inside an urban teaching hospital. Therefore, this study aims to collate the experiences of patients on dialysis and present results for this group in aggregate. The Picker Principles of Patient Centered Care is used to guide this research because they are accepted globally as a framework to understand patient experiences in healthcare in both research and quality improvement projects.^21^^,^^15^ The Standards for Reporting Qualitative Research were used to guide this work.^26^

### Utilizing narratives

In general, examining narratives with qualitative research methods captures the points of view of other people without predetermining those views through prior selection of questionnaire categories.^27^ An individual's personal narrative can better highlight aspects of care that may trigger strong emotions and the words they use to describe the relationship between their health services and their outcomes. Thus, rather than collating patient experiences using other popular methods such as a survey, this study highlights stories and experiences among patients who have complex medical conditions, who access various health services and seek care from many healthcare professionals and teams.^28^ This approach may offer important insights into healthcare improvements from the patient perspective.

### Participants

Eligible patients were English-speaking adults at least 18 years old who have received hemodialysis at this outpatient clinic for at least three months and have at least one medication prescribed and monitored by the clinic. Any patients meeting eligibility criteria between May 1, 2023 and Aug 31, 2023 were approached by a social worker and the primary researcher (TZ) during the patient's hemodialysis appointment. Convenience sampling was employed to reduce bias and maximize participant recruitment. The social worker introduced the eligible patient to TZ who then provided a brief verbal description of the study and patients were invited to contact the research team if they were interested in participating. If interested, written participant consent was obtained by TZ and an interview was scheduled during a future hemodialysis appointment at the participants' convenience. Patients were recruited and data were collected and analyzed concurrently, and continued until data saturation was reached, where no new themes emerged. While the research team initially aimed to recruit 13 participants based on a methodologically similar study that examined the perspectives of patients on hemodialysis towards medication self-management,^29^ recruitment was terminated after nine participants as the research team discussed and determined through consensus that saturation of data was reached.

### Data collection and analysis

The interview guide was developed by TZ and questions were focused on processes, including a typical day of taking medications or a medication change. The Picker Principles^21^ were used to develop probing questions regarding continuity of care and involvement of various individuals (HCPs, caregivers, self). These questions were reviewed and revised by the hospital's patient ambassadors who have experience working with patients on hemodialysis to establish face validity. Patient ambassadors are volunteers who have personally been involved with kidney transplant and have received specific training to speak with patients on hemodialysis regarding their experiences and provide education and information regarding kidney transplant. Thereafter, minimal amendments were made to ensure patient understanding. See Appendix A for interview guide.

Interviews were conducted by the primary researcher, TZ, a female pharmacist (BScPhm) and graduate student. Participants were made aware of the primary researcher's profession and work experience, and that the study was part of the researcher's graduate work. Co-investigator, MM, is a pharmacist who has experience caring for patients on hemodialysis. Co-investigator AA has experience conducting qualitative research related to PCC. There was no prior relationship between study participants and researchers. Interviews were recorded on a one-way Microsoft Teams call utilizing the transcription tool. Interviews were then transcribed verbatim by TZ and co-investigator, AA. Patient demographics including age, sex, dialysis vintage, length of time at the TGH dialysis clinic, medical conditions, home medications and community pharmacy were collected from the patient's chart after each interview was conducted. See Appendix B for data collection sheet.

Data were analyzed using a general inductive approach. The Picker Principles of Patient-Centered Care were used as a lens through which the data were analyzed. Through discussions, the research team established consensus regarding how each emerging theme corresponded to the Picker Principles. After six interviews were conducted, transcripts were reviewed several times, and an initial round of coding was conducted by TZ. A random sample of three transcripts was provided to co-investigator MM, a pharmacist who also has experience working with patients on hemodialysis, for independent coding. After the initial coding phase, TZ and MM met to reach consensus on themes and developed a coding tree. Subsequent interviews were conducted one-by-one and analyzed using this coding tree and emerging themes were noted. Amendments to the coding tree and emerging themes were made iteratively through consensus by MM and TZ after each interview. AA, a member of the research team who is not a clinician, also reviewed the themes identified by MM and TZ and further amendments were made through consensus by all three research team members. No new themes were noted to have emerged after nine interviews. See Appendix C for data extraction table. To enhance the trustworthiness of the data, TZ underwent peer debriefing with other multidisciplinary HCPs caring for patients on hemodialysis, including social workers, dietitians and other pharmacists, to ensure TZ's experiences were similar and generally representative of patients on hemodialysis. Finally, TZ performed reflexive journaling to reflect on her inherent bias as a HCP before and after each interview. While peer debriefing revealed that TZ's interview experiences were similar and generally representative of patients on hemodialysis to other HCPs, reflexive journaling helped identify how TZ approached earlier interviews with an information-gathering focus, often used by HCPs to create care plans, rather than an experience-gathering focus, used by researchers to elicit participant narratives. This led to a change in awareness and questioning style by TZ for later interviews.

## Results

**Fig. 1:**
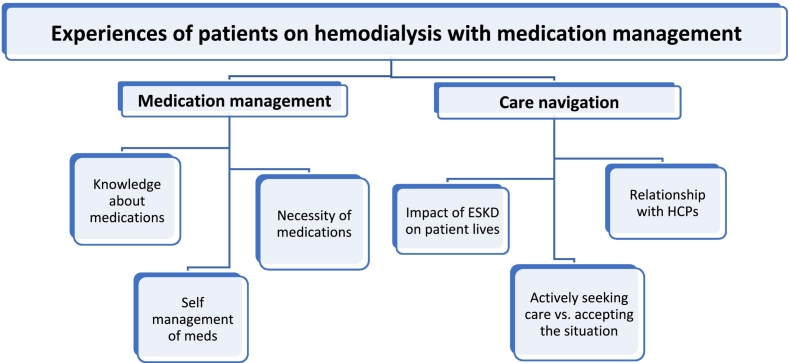
Coding tree.

Two major themes, medication management and care navigation, were identified along with several subthemes for each major theme discussed in detail below. See Fig. 1 for coding tree. A total of nine patients with a mean age of 71 years old who have been on hemodialysis for an average of 8 years were interviewed between May and July 2023. Interviews ranged from 9 to 38 min and lasted an average of 27 min. Seven of the nine patients were male and most had three or more medical conditions, most commonly hypertension (*n* = 8) and dyslipidemia (*n* = 6) in addition to ESKD. On average, patients took 11 medication doses per day, with some medications taken once daily and others up to three times daily. Most patients had their medications filled at a chain pharmacy rather than an independent pharmacy. See Table 1 for patient characteristics.

**Table 1:** Participant characteristics.

Demographics	*n* = 9 (%)
Age group	
<50	0 (0)
50–60	2 (22)
61–70	3 (33)
71–80	2 (22)
>81	2 (22)

Male sex	
M	7 (78)

Dialysis vintage	
<1 year	0 (0)
1–3 years	3 (33)
4–6 years	2 (22)
>7 years	4 (44)

No. of medical conditions in addition to ESKD	
≤3	1 (11)
>3	8 (89)

### Medication management

All patients demonstrated they had extensive knowledge about medications stemming from their experiences with taking medications for various chronic conditions. In addition to their knowledge of medications, patients discussed experiences related to self-managing medications. Finally, patients expressed that taking and managing their medications is a necessity to their well-being and survival.

#### Knowledge about medications

Patients spoke with confidence about what medications they were taking or had taken in the past. They provided details about dosages, how to take these medications, what they were prescribed for, and side effects they experienced. Patients noted that they acquired medication knowledge through their various experiences with healthcare and illness and through HCPs' explanations of medications, including the purpose and potential adverse effect.

“They prescribed me the bisoprolol because my heart rate is a little bit fast. Right now, I'm taking 2.5[mg], but that time I have to put it again around 1.25[mg] only because sometimes it's too low. But now, 2.5 is okay. Stable. Before when I'm not taking it, I don't feel good because the heart, my pulse rate is really fast. But when I took it, oh it's better, a lot better” (Patient 1, 68-year-old male).

“In the aftermath of [organ] transplant, one of the things that they do is to, you know, they come in with your medication, the nurse does, and they go over with you again and again and again. You're taking this for this. This is this drug. You're taking this for this. So you know which is the cyclosporine… and what the purpose of all the medications is” (Patient 7, 68-year-old male).

#### Self-management of medications

Patients further spoke about how they manage their medications, including obtaining newly prescribed medications, coordinating refills, and remembering to take medications. Patients described how they took on the task of medication management as their own responsibility.

“I've gotta keep an eye on everything, you know? See what I'm running out of, what I need. I just, you know need to keep record according to what I have” (Patient 3, 63-year-old male).

“My wife's dead, but I'm the only one who's doing it. Everything. In the morning, I have to put it in the medication [box]. Monday, Wednesday, something like that, you know? Everyday I do it, breakfast, lunch, in the evening, yeah. Everyday, everyday [chuckles]” (Patient 1, 68-year-old male).

Patients spoke about the barriers and facilitators they encountered when self-managing medications. Barriers include forgetting to take medications, especially when not feeling well, and coordinating refills, particularly for long-term medications that often change in dosage. Facilitators include utilizing blister-pack programs offered by pharmacies to help organize medication scheduling, supply and delivery.

“My only problem with medications is that, especially in the evenings, I will get my medication ready to take and then I'll fall asleep and then I wake up three hours [later] and you're supposed to take it in a timely manner…so every once in a while, I have one of those” (Patient 7, 68-year-old male).

“It's tough just keeping track of everything, you know what I mean. I'm constantly just trying to… I wish I could just start from Ground Zero with the pharmacy and get all my meds at one time, so that I'm ordering everything every three months, so I'm getting three months-worth of everything, you know, instead of having to run back to the pharmacy every month. Yeah, it's quite tedious as well, right?” (Patient 3, 63-year-old male).

“Of course, the drug stores are getting more sophisticated, and Rexall put all my drugs, morning, noon, night into little capsules. And my dosages are all planned out every month. And we pick them up or get them delivered and I don't have any problems managing those” (Patient 5, 88-year-old male).

#### Necessity of medications

Patients expressed that they were motivated to practice good medication management and adhere to their medications as prescribed as these practices were essential to their wellbeing and survival.

“Then from day one, it's like OK you have to learn this. You do have to learn this. And so yeah. Well, I'm not done yet. OK, then it's like, you have this disease, and you're gonna die. And here's your only hope. So anyway, that gets you so you put your house in order” (Patient 7, 68-year-old male).

“I have to take them. So I don't have no problem with it because if the doctor prescribed something for me. I usually take my stuff. That's one thing with me. I take my medication… Like I said, I listened to the doctors. I do what they tell me to do… I'm not ready to die. I need to still survive.” (Patient 6, 58-year-old female).

### Care navigation

In addition to speaking about medication management, patients shared stories about their experiences navigating healthcare while on dialysis. Patients spoke about the impact of ESKD on their lives. They highlighted times when they felt it was necessary to strongly advocate for themselves to receive appropriate care versus times when they felt they had to accept their health status. Finally, patients discussed their relationships with HCPs while navigating care, including building good rapport with those considered to be gatekeepers to good care, compared to experiences with HCPs that left them feeling wary about their care.

#### Impact of ESKD on patient lives

Patients shared their experiences with feeling unwell or being hospitalized for reasons related to having ESKD. Patients described lifestyle challenges associated with ESKD and hemodialysis such as having to adhere to a strict diet, and managing side effects, such as cramping, pain and extreme fatigue.

“The only thing with dialysis now, is the cramping. That's the only thing. That's the only thing that it's, you know, I try you know, I try. But that is the only one I can't beat up till now. But I forever trying, you know” (Patient 4, 87-year-old male).

“I mean one thing which I fight… fought is depression. I think everybody has, but mine normally do not last more than 24 hours. I bounce back. I mean, I get upset, you know, I mean. Yeah, but we'd like to go on cruises, but I still could. They have dialysis at sea, but ah. We used to travel so much and I miss it. And my wife just went to Europe with my daughter-in-law, and they had a wonderful time. I don't mind her going away but, you know, I can only go away for overnight. You know” (Patient 5, 88-year-old male).

#### Actively seeking care vs. accepting the situation

Throughout their experiences with navigating care, many patients mentioned that there were times they had to strongly advocate for themselves to have their care needs met, and times where they felt they had to accept their situation.

“It's even a little bit more diverse than that. I'm dealing with the dermatology team. I'm dealing with my lung transplant team and I'm dealing with a nephrology team. And there have been occasions where left hand and right hand are not in contact but that is up to me to say ok, you're not. You're not communicating or this has been overlooked, or I was told this by these people. You didn't get the message. What's going on”… “I have learned how to be my own advocate in this whole process” (Patient 7, 68-year-old male).

“Well if a new medication is not okay, I have to tell it to the one who prescribed it to me that it's not good for me” (Patient 1, 68-year-old male).

Acceptance was often accompanied with a sense of resignation, where the patient had grappled with their health situation and concluded that there may not be another alternative other than acceptance.

“I'll just put up with the arthritis. Yeah. [chuckles] So that's what I do. If it gets too bad I just take normal Tylenol now. But it's worse like when it rains, it's, for me is the pain time” (Patient 9, 58-year-old male).

“After a while [the nausea] goes away, Yeah, but you see, you cannot control it. That's the only medication that I remember, oh god, I don't like it, but I have to do it. I have to take it… but I mean it just is one episode a day, something like that, and then after a while it's gone. I didn't brought it up [with my doctor] because it's going away. It's not all day long you suffer from it, no. Just after that and its, you're okay” (Patient 1, 68-year-old male).

#### Relationships with healthcare providers

Patients shared perspectives about their relationships with various HCPs. Patients noted that they turned to certain HCPs who acted as connectors to care if they felt ill or had questions about their medications or other chronic conditions. Often, patients saw these HCPs regularly at scheduled appointments or meetings, or during hemodialysis treatments.

“I normally deal with the doctors here, particularly Nurse Practitioner, just because she normally really makes sure things are done, you know, I don't have to, you know, double check with her. She's normally very, very good so I kind of tend to gravitate towards her” (Patient 3, 63-year-old male).

“Well, the main thing is for me that makes it easy is that the transplant outpatient clinic care in the hospital is fabulous. And so they are very good at coordinating with your doctors to make sure you medications list is up to date” (Patient 7, 68-year-old male).

Patients did not build good rapport with all HCPs they encountered. Some patients voiced strong opinions and previous negative experiences with HCPs. Patients spoke of times they were not open to advice about medication changes from HCPs because the HCPs were not part of their usual care team or did not acknowledge their experiential knowledge. Patients also spoke of frustrating encounters with HCPs who were difficult to reach or coordinate care with.

“When I tell people that I come down here to do the dialysis [chuckles], they say isn't there a dialysis place at [another closer] hospital? I said, I'm not going there. Because I even remember with my foot, uhm, after they told me they couldn't see anything… You are not my transplant doctor. Why am I increasing [the dose]? To harm myself? I just go to you to see what's going on with my foot and I'm not gonna increase no medication. You are not my doctor for my transplant so I would never do it. Some people would, not me. I have to come to my doctor to do that. So, I didn't. I didn't shrink it; I didn't increase it” (Patient 6, 58-year-old female).

## Discussion

Through qualitative interviews, this study reveals experiences related to the complexities of medication management and navigating outpatient care among nine patients receiving hemodialysis at a clinic inside an urban teaching hospital. Patients highlighted knowledge of their medications acquired through their experience taking multiple medications and through interactions with healthcare providers. Although patients expressed challenges with self-managing medications, including the cumbersome process of coordinating refills and remembering to take their many medications, most were motivated to manage their medications well as they viewed it as essential to their wellbeing and survival. Given the impact of ESKD on these patients' lives, including long hospital stays and side effects of hemodialysis, patients expressed that they had to strongly advocate to have their healthcare needs addressed and sometimes they felt resigned to accept their health situation. When asked about their experience navigating care with several different HCPs, patients noted they had good rapport with select HCPs who were attentive to their needs and helped them navigate the healthcare system. Through understanding these experiences, this study reveals opportunities where patient-centered care may be improved for patients on hemodialysis, including support for self-care in regard to medication management and strengthening IPC.

This study adds to the body of work evaluating the key principles of PCC that have been shown to be important across different patient populations in order to enhance patient satisfaction, health outcomes, and reduced utilization of care services.^30^^,^^31^ Similar to Mills et al.'s findings of key principles of PCC in patients seeking dental care, patients in this study did not discuss involvement of family and carers or the importance of physical or environmental needs.^30^ Moreover, like Van Shalkwijk et al.'s study of patients with non-obstructive coronary artery disease, patients spoke of the challenges associated with navigating multiple institutional or clinic visits and repetitive testing just to confirm a diagnosis and be connected to a specialist. Van Shalkwijk et al. also noted the importance of having a main point of contact, often a nurse practitioner, to help patients coordinate care and communicate information related to medications and their side effects.^31^ Although each Picker Principle highlights an important component of PCC, findings in this study indicate that not all principles may be of equal importance to patients with different health situations and care needs.

The patients in this study emphasized two main Picker Principles of Patient-Centered Care: clear information, communication and support for self-care, and effective treatment by trusted professionals. Addressing ways to enhance these two Picker Principles specifically may provide insight on how to improve patient-centered care for patients on hemodialysis. The Picker Principle of clear information, communication and support for self-care stipulates that patients should receive high quality and accessible information at every stage of their journey. This information should be provided at appropriate times, be understandable and support patients in managing their care.^21^ Parker et al. revealed that patients who were supported in self-managing their medications seemed to be better at acquiring the resources they needed to meet their healthcare needs.^29^ This reaffirms the importance of providing good support for self-care. Furthermore, the challenges that patients on hemodialysis encountered in navigating and coordinating care while interacting with multiple care providers has been previously described by Allen et al. and Roberti et al.^32^^,^^33^ These studies also found that much of the burden of treatment that patients with chronic kidney disease take on involves navigating care and managing complex medications.^32^^,^^33^

Though patients in this study were motivated to engage in good medication management, patients felt this to be an essential component for survival, and thus a willingness to shoulder the burden of self-management does not mean patients actually feel supported to do so. This raises an important question of whether patients are receiving support from HCPs for self-management of their medications. Contrary to Parker et al., this study did not emphasize the importance of involvement of family and carers.^29^ Patients in this study did not speak of the role of wider support networks in their overall care despite being asked. While this may not mean that the support of family and carers was completely absent or irrelevant, it may mean that patients truly see medication management and care navigation as their own responsibility and burden. If this is the case, perhaps even more thought should be placed on how to better support patients in self-management of medication, reducing challenges associated with care coordination and providing clear information at every stage of their dynamic journey as a patient on hemodialysis. While adequately supporting patients for self-care may be important in reducing barriers associated with medication management, this may be difficult to achieve in practice. Given the number of HCPs involved, there may be a lack of role clarity and poor communication as to who is responsible for supporting patients in various aspects of self-care. Pharmacists, being medication experts, may be well positioned to take on this role as it pertains to self-management of medications. Further research on the experiences of patients on hemodialysis with respect to their medication management is needed to fully understand the challenges of self-management and strategies to improve support for self-care.

In addition to support for self-care, patients in this study also emphasized the importance of effective treatment by trusted professionals. This Picker Principle stipulates that positive therapeutic relationships are at the heart of PCC. Care should not only be clinically appropriate and effective but should also meet the individual needs of patients.^21^ Patients in this study highlighted the need to improve care fragmentation, which is defined as “healthcare delivery that is spread out across an excessively large number of poorly coordinated providers”.^34^ Given that patients relied on select HCPs that they had good rapport to help coordinate care with other providers, this indicates an opportunity for improved interprofessional collaboration through care coordination lead by one, or select HCPs, as a component of reducing the burden of care navigation that patients on hemodialysis face. This study uniquely highlights the importance of good interprofessional collaboration to patients based on their experiences. Many existing studies that examine interprofessional collaboration and collaborative practice focus on the experiences of healthcare providers rather than patients.35., 36, 37, 38 In a recent scoping review, Morgan et al. found only seven studies examining patient experiences with interprofessional collaboration.^38^ This scoping review identified a variety of interprofessional, collaborative activities, which included monthly interprofessional case conferences and interprofessional diabetes education and indicated that patients felt greater engagement in self-management of their conditions.^38^ Other components of PCC such as involvement in decisions and shared decision making were also noted.^38^ Patients in our study spoke positively regarding instances where HCPs engaged in collaborative care with other providers. This included times when HCPs helped patients coordinate appointments with specialists or provided timely prescription renewals directly to a patient's pharmacy. Given the importance of self-management patients on hemodialysis voiced in our study, this may be another reason why efforts to improve interprofessional collaboration should be considered to reduce the burden of treatment patients experience and address care fragmentation. Further exploration of a model of interprofessional collaboration that includes a HCP care coordinator may be warranted. Additionally, more studies in the field of interprofessional collaboration research should adopt a patient-centered approach to reveal aspects of care that truly matter to patients.

This study has several strengths and limitations. One limitation is that eligibility criteria may have excluded certain groups within the general ambulatory hemodialysis population. However, criteria were selected based on the limited scope and resources available to the study. Patients included were mostly male and all patients were 50 years or older, thus the sample may not represent experiences of patients on hemodialysis who are younger or female. The participants in this study might represent a self-selected sample of individuals who were open to speak about their experiences regarding medication management and care navigation. These individuals may hold experiences that differ from other patients on hemodialysis who elected not to participate. Furthermore, two members of the research team are pharmacists and may carry intrinsic bias on the definition and value of medication management, care navigation and PCC. Input from a non-clinical member of the research team was valuable to minimize potential bias. While study findings cannot be generalized to all ambulatory patients on hemodialysis, participants did take an average of 11 pills per day and most had three or more chronic medical conditions, which is similar to other reports of patients on hemodialysis in the literature. Another strength is that the Picker Principles of Patient Centered Care was used as a guide to analyze data, helping to ground this work in a PCC framework.

## Conclusions

This study revealed that patients on hemodialysis face challenges to managing their complex health needs, including medication management, and may not be well supported due to a fragmented healthcare system, despite being knowledgeable and motivated to engage in good self-management of their medications. There is a pressing need for clear and timely information from trusted HCPs, support for self-care, and enhanced interprofessional collaboration to optimize medication management practices and reduce care fragmentation. This study further highlights opportunities for addressing IPC and improving care for patients on hemodialysis, including introducing a HCP care coordinator model in hemodialysis care. Further studies should investigate strategies or develop interventions to improve self-management and interprofessional collaboration on the experiences of medication management for patients on hemodialysis. Given that patients on hemodialysis currently have select HCPs who help them coordinate fragmented care, future studies should investigate ways in which more HCPs can support and facilitate care coordination roles through effective interprofessional collaboration, including taking on roles as care coordinators connected to patients in this capacity more formally. Furthermore, patients on hemodialysis are not the only group of patients who take many medications and must engage with navigating a fragmented healthcare system. Further studies are needed to characterize the unique experience of other groups, such as geriatric patients or patients who have undergone organ transplantation, to reveal ways to improve PCC for these groups.

## Disclosures

We confirm that this work is original and has not been published elsewhere, nor is it currently under consideration for publication elsewhere, and its publication is approved by all authors and tacitly or explicitly by the responsible authorities where the work was carried out, and that. If accepted, this work will not be published elsewhere in the same form, in English or in any other language, including electronically without the written consent of the copyright-holder.

## Funding

This research did not receive any specific grant from funding agencies in the public, commercial, or not-for-profit sectors.

## CRediT authorship contribution statement

**Tracy Zhang:** Writing – review & editing, Writing – original draft, Visualization, Validation, Project administration, Methodology, Investigation, Formal analysis, Data curation, Conceptualization. **Mai Mohsen:** Writing – review & editing, Validation, Methodology, Formal analysis, Conceptualization. **Angelina Abbaticchio:** Writing – review & editing, Validation, Methodology, Formal analysis. **Marisa Battistella:** Writing – review & editing, Visualization, Validation, Supervision, Project administration, Methodology, Formal analysis, Data curation, Conceptualization.

## Declaration of competing interest

The authors declare that they have no known competing financial interests or personal relationships that could have appeared to influence the work reported in this paper.

## Figures and Tables

**Table:** 

Participant ID/sex	Year of birth	HD vintage	Time at TGH HD clinic	Medical conditions	Home medications	Community pharmacy
						
						
						

**Table:** 

Theme: medication management	
Subtheme	Exemplar quotes
Knowledge about medications	“They prescribed me the bisoprolol because my heart rate is a little bit fast. Right now, I'm taking 2.5[mg], but that time I have to put it again around 1.25[mg] only because sometimes it's too low. But now, 2.5 is okay. Stable. Before when I'm not taking it, I don't feel good because the heart, my pulse rate is really fast. But when I took it, oh it's better, a lot better.” (Patient 1, 68-year-old male) “I've been on a thyroid pill for 40 years and they are increasing the strength because they think it's making me sleepy… the change they are making which is to increase the strength of my thyroxine; thyroid medication. That's that hasn't taken effect yet but they just did that a week ago.” (Patient 5, 88-year-old male) “In the aftermath of [organ] transplant, one of the things that they do is to, you know, they come in with your medication, the nurse does, and they go over with you again and again and again. You're taking this for this. This is this drug. You're taking this for this. So you know which is the cyclosporine… and what the purpose of all the medications is.” (Patient 7, 68-year-old male) “Prednisone is not a good medication. I had problems with it. Both of my feet. My toe was broken… I know the Prednisone did that to my foot because that's what [the doctor] said.” (Patient 6, 58-year-old female)
Self-management of medications	“I've gotta keep an eye on everything, you know? See what I'm running out of, what I need. I just, you know need to keep record according to what I have” (Patient 3, 63-year-old male) “My wife's dead, but I'm the only one who's doing it. Everything. In the morning, I have to put it in the medication [box]. Monday, Wednesday, something like that, you know? Everyday I do it, breakfast, lunch, in the evening, yeah. Everyday, everyday [chuckles]” (Patient 1, 68-year-old male) “I take 5 different pills in the morning time. Evening time before bed I take one. Lunch time I supposed to take 1 [pill]. But when I home I take it. When I not home, I don't.” (Patient 5, 88-year-old male) “I normally, you know, get up in the morning. I have a certain set of meds I take and then in the evening I have a certain, you know medications to take, and sometimes, when I'm not doing good, I might forget to take it.” (Patient 3, 63-year-old male) “My only problem with medications is that, especially in the evenings, I will get my medication ready to take and then I'll fall asleep and then I wake up three hours [later] and you're supposed to take it in a timely manner…so every once in a while, I have one of those.” (Patient 7, 68-year-old male) “It's tough just keeping track of everything, you know what I mean. I'm constantly just trying to… I wish I could just start from Ground Zero with the pharmacy and get all my meds at one time, so that I'm ordering everything every three months, so I'm getting three months-worth of everything, you know, instead of having to run back to the pharmacy every month. Yeah, it's quite tedious as well, right?” (Patient 3, 63-year-old male) “Of course, the drug stores are getting more sophisticated, and Rexall put all my drugs, morning, noon, night into little capsules. And my dosages are all planned out every month. And we pick them up or get them delivered and I don't have any problems managing those.” (Patient 5, 88-year-old male)
Necessity of medications	“Then from day one, it's like OK you have to learn this. You do have to learn this. And so yeah. Well, I'm not done yet. OK, then it's like, you have this disease, and you're gonna die. And here's your only hope. So anyway, that gets you so you put your house in order” (Patient 7, 68-year-old male) “I have to take them. So I don't have no problem with it because if the doctor prescribed something for me. I usually take my stuff. That's one thing with me. I take my medication… Like I said, I listened to the doctors. I do what they tell me to do… I'm not ready to die. I need to still survive.” (Patient 6, 58-year-old female)

Theme: care navigation	
Subtheme	Exemplar quotes
Impact of ESKD on patient lives	“I mean I had a big set-back. Somehow, my [dialysis] line got compromised, I don't know how. And I got this disease you know, the blood disease. So I now have to take antibiotics for the rest of my life. They never could locate all of it inside me. They opened me up and I was in the hospital for 2 months, I was in ICU for two months.” (Patient 5, 88-year-old male) “I had my transplant, and I didn't feel well most of the time. Since I had transplant, started dialysis last year, I was admitted here [in the hospital] two or three times. First one was because, I didn't start the dialysis yet, but it was in 2022. Yes, they admitted me, and they drained the fluid out. Yeah, that hurt. Yeah, because they didn't like, put me to sleep or anything.” (Patient 6, 58-year-old female) “The only thing with dialysis now, is the cramping. That's the only thing. That's the only thing that it's, you know, I try you know, I try. But that is the only one I can't beat up till now. But I forever trying, you know.” (Patient 4, 87-year-old male) “I mean one thing which I fight… fought is depression. I think everybody has, but mine normally do not last >24 h. I bounce back. I mean, I get upset, you know, I mean. Yeah, but we'd like to go on cruises, but I still could. They have dialysis at sea, but ah. We used to travel so much and I miss it. And my wife just went to Europe with my daughter-in-law, and they had a wonderful time. I don't mind her going away but, you know, I can only go away for overnight. You know.” (Patient 5, 88-year-old male)
Actively seeking care vs. accepting the situation	“It's even a little bit more diverse than that. I'm dealing with the dermatology team. I'm dealing with my lung transplant team and I'm dealing with a nephrology team. And there have been occasions where left hand and right hand are not in contact but that is up to me to say ok, you're not. You're not communicating or this has been overlooked, or I was told this by these people. You didn't get the message. What's going on”… “I have learned how to be my own advocate in this whole process” (Patient 7, 68-year-old male) “Well if a new medication is not okay, *I* have to tell it to the one who prescribed it to me that it's not good for me.” (Patient 1, 68-year-old male) “I'll just put up with the the arthritis. Yeah. [chuckles] So that's what I do. If it gets too bad I just take normal Tylenol now. But it's worse like when it rains, it's, for me is the pain time” (Patient 9, 58-year-old male) “Yup, I sort of accept it. You know, all my life I never went near a hospital until I was 70. I had both my hips and both my knees replaced within three months. But that was a shock.” (Patient 5, 88-year-old male) “You want to do but you can't do. But other than that, everything is cool these days, you know? You have to learn to accept [chuckles]” (Patient 4, 87-year-old male) “After a while [the nausea] goes away, Yeah, but you see, you cannot control it. That's the only medication that I remember, oh god, I don't like it, but I have to do it. I have to take it… but I mean it just is one episode a day, something like that, and then after a while it's gone. I didn't brought it up [with my doctor] because it's going away. It's not all day long you suffer from it, no. Just after that and its, you're okay.” (Patient 1, 68-year-old male)
Relationship with healthcare providers	“I normally deal with the doctors here, particularly Nurse Practitioner, just because she normally really makes sure things are done, you know, I don't have to, you know, double check with her. She's normally very, very good so I kind of tend to gravitate towards her” (Patient 3, 63-year-old male) “Well, the main thing is for me that makes it easy is that the transplant outpatient clinic care in the hospital is fabulous. And so they are very good at coordinating with your doctors to make sure you medications list is up to date.” (Patient 7, 68-year-old male) “When I tell people that I come down here to do the dialysis [chuckles], they say isn't there a dialysis place at [another closer] hospital? I said, I'm not going there. Because I even remember with my foot, uhm, after they told me they couldn't see anything… You are not my transplant doctor. Why am I increasing [the dose]? To harm myself? I just go to you to see what's going on with my foot and I'm not gonna increase no medication. You are not my doctor for my transplant so I would never do it. Some people would, not me. I have to come to *my* doctor to do that. So, I didn't. I didn't shrink it; I didn't increase it.” (Patient 6, 58-year-old female) “I know my body, I know how it feels, you know you can say whatever you want to the doctor, but you know when you feel, you know, when your [blood] pressure drops, you know, you can really feel that right. Like I know when it's good and I know when it's coming down and stuff because I'm actually feeling it. You know, you know the doctor can look but he, you know, you know, he would have no clue on how the feelings he's gotta go off of.” (Patient 3, 63-year-old male) “My GP… I switched [all my prescriptions] to them… Except for the dermatologist. But that's not good because the dermatologist never answers his phone, never answers emails. And you know just, its like pulling teeth. For an appointment. For one hour. But most of my meds now I can phone up [my GP] and get them renewed.” (Patient 5, 88-year-old male) “A couple of weeks ago I had a number of surgeries and because that doctor is not just here, but also practices in other settings there have been a number of times where there's been sort of issues that he, you know, hasn't been in contact with. I've just learned that I really have to be clear about that.” (Patient 7, 68-year-old male)

## Appendices

### Appendix A. Interview guide

Note to participant: During our discussion in this interview, in order to maintain privacy, if you need to refer to other individuals I ask that you please refrain from using personal identifying information such as their name. It may be easiest to refer to their roles (ie – nurse, sister, etc).

I know as a patient on hemodialysis, you probably take a lot of medications. I'm really interested in hearing more about your experiences managing these medications. Based on my limited experience, I gather that there are probably lots of other individuals who have a role to play in your medication management – different doctors or nurse practitioners who prescribe things, pharmacy teams, people in hemodialysis, people in other specialty clinics etc. With having to navigate all of that and also remember what to take and when, I'm curious to know what that's like and to hear your stories and experiences with all of it. I have some questions here that we can go through to help us get the conversation started…

1. What does a typical day when you have hemodialysis look like for you?
  a. Please map out when you take your medications, who helps with care, other healthcare appointments you may have or healthcare providers you may see.
    i. Ask about people involved: Who are the healthcare providers you see? How do you interact with them? Who are other caregivers who may be involved in helping you on these days?
    ii. Are there things that you particularly like about these days? Or dislike?
  b. How do you feel after one of these days?
2. What does a typical day when you don't have hemodialysis look like for you?

a. Please map out when you take your medications, who helps with care, other healthcare appointments you may have or healthcare providers you may see.
  i. Ask about people involved: Who are the healthcare providers you see? How do you interact with them? Who are other caregivers who may be involved in helping you on these days?
  ii. Are there things that you particularly like about these days? Or dislike?
b. How do you feel after one of these days?

3. Comparing and contrasting hemodialysis vs. non-hemodialysis days, are there changes to your medication or healthcare routine between these two days?

a. Does anybody help you manage these changes?

4. Thinking about your last medication change:
  a. Please walk me through what happened: who made the change, why, how you felt about it, and the steps involved with how or if the medication change was implemented
  b. How did you feel the process went from start to finish?
    i. Did you agree with the need for a change?
    ii. Was it easy to understand the change?
    iii. Was it easy to obtain the medication?
  c. What healthcare providers were involved in this process? What other carers/family/friends were involved?
  d. How do you feel now that the change has been implemented?
5. Thinking about the last time you were prescribed a new medication:
  a. Please walk me through what happened: who made the change, why, how you felt about it, and the steps involved with how or if the medication change was implemented
  b. How did you feel the process went from start to finish?
    i. Did you agree with the need for a new prescription?
    ii. Was it easy to understand what the new medication would do? How to take it? Side effects?
    iii. Was it easy to obtain the medication?
  c. What healthcare providers were involved in this process? What other carers/family/friends were involved?
  d. How do you feel now that the change has been implemented
